# In Vitro Study of the Cytotoxic, Cytostatic, and Antigenotoxic Profile of *Hemidesmus indicus* (L.) R.Br. (*Apocynaceae*) Crude Drug Extract on T Lymphoblastic Cells

**DOI:** 10.3390/toxins10020070

**Published:** 2018-02-06

**Authors:** Eleonora Turrini, Cinzia Calcabrini, Massimo Tacchini, Thomas Efferth, Gianni Sacchetti, Alessandra Guerrini, Guglielmo Paganetto, Elena Catanzaro, Giulia Greco, Carmela Fimognari

**Affiliations:** 1 Department for Life Quality Studies, Alma Mater Studiorum, University of Bologna, Corso d’Augusto 237, 47921 Rimini, Italy; eleonora.turrini@unibo.it (E.T.); cinzia.calcabrini@unibo.it (C.C.); elena.catanzaro2@unibo.it (E.C.); giulia.greco9@unibo.it (G.G.); 2 Department of Life Sciences and Biotechnology, University of Ferrara, Piazzale Luciano Chiappini 3, Malborghetto di Boara, 44123 Ferrara, Italy; massimo.tacchini@unife.it (M.T.); gianni.sacchetti@unife.it (G.S.); alessandra.guerrini@unife.it (A.G.); guglielmo.paganetto@unife.it (G.P.); 3 Department of Pharmaceutical Biology, Institute of Pharmacy and Biochemistry, Johannes Gutenberg University, 55128 Mainz, Germany; efferth@uni-mainz.de

**Keywords:** *Hemidesmus indicus*, cancer cells, apoptosis, cell cycle, genotoxicity, antigenotoxicity

## Abstract

In traditional Indian medicine, the crude drug *Hemidesmus indicus* root—commonly known as Indian sarsaparilla—is used alone or in poly-herbal preparations for the treatment of a wide range of diseases. The present study focuses on the cancer chemopreventive and therapeutic potential of *H. indicus* extracts on an acute lymphoblastic leukemia cell line (CCRF-CEM). With this aim in mind, we subjected *H. indicus* roots to two subsequent extractions (hydro-alcoholic extraction and soxhlet extraction). As DNA damage is an important prerequisite for the induction of mutations/cancer by genotoxic carcinogens, cancer chemoprevention may be achieved by preventing genotoxicity. Through an integrated experimental approach, we explored the genoprotective potential of the soxhlet *H. indicus* extract against different mutagenic compounds and its cytotoxic, proapoptotic, and cytostatic properties. In our experimental conditions, *H. indicus* induced a cytotoxic effect involving the activation of both intrinsic and extrinsic apoptotic pathways and blocked the cell cycle in the S phase. Moreover, the antigenotoxicity results showed that the extract was able to mitigate DNA damage, an essential mechanism for its applicability as a chemopreventive agent, via either the modulation of extracellular and intracellular events involved in DNA damage. These data add to the growing body of evidence that *H. indicus* can represent a noteworthy strategy to target early and late stages of cancer.

## 1. Introduction

From ancient times to recent times, natural products have always represented a keystone in medicine. Shamans and early physicians empirically learnt the power of herbal medicine, while the advance in medical knowledge helped promote the understanding and demonstration of the pharmacologic basis of where that power lies. Now, it is clear that the complexity of natural products is the key of their powerful activity. Natural products store within them numerous molecules, all potentially pharmacologically active, which together can affect more than one biological pathway, resulting in a wide global activity [1]. In this context, the potentiality of natural drugs to treat a complex pathology as cancer is enormous.

Carcinogenesis is a multistep process characterized by subsequent phases that foresees the transformation of normal cells into neoplastic ones [2]. This process starts with the acquisition of DNA mutations in different key genes (oncogenes), named the initiation phase. When the same mutated cells clonally expand, they go through promotion phase that results into progression when the malignant phenotype is acquired and the frank tumor generated [2]. Each single phase of this process represents a possible target for intervention, and the identification and characterization of agents capable of reversing, retarding, or inhibiting the carcinogenic process has the same therapeutic value as finding a drug able to eradicate full-blown cancers. Indeed, the World Health Organization indicated the prevention of breast cancer as a factual, therapeutic approach [3,4].

Natural products can intervene in the early stages of carcinogenesis, acting as chemopreventive agents, with the late ones acting as chemotherapeutic agents. Resveratrol, obtained for example from the root of *Polygonum cuspidatum*, sulforaphane, synthesized in different organs of plant species belonging to *Brassicaceae* family, and epigallocatechin gallate from green tea are only three examples of natural compounds with chemopreventive and antitumor properties, demonstrated by in vitro and in vivo studies [5,6,7]. Moreover, it has been confirmed that the antitumor potential of natural products cannot always be ascribed to a single phytochemical, but to the combination of the many active molecules that synergistically point at restraining different targets. For instance, the two major cytotoxic terpenes of the essential oil obtained from the ripe fruits of *Echinophora spinosa* L. (*Apiaceae*) did not exhibit the same antiproliferative effect of the phytocomplex essential oil toward human U937 promonocytoid cells. Only the addition to the mixture of a third terpene, *per se* not toxic, recapitulates multiple salient features of the essential oil’s cytotoxicity [8]. In the same way, the combination of three main *Angelica sinensis* phthalides is not sufficient to sum up the anti-tumor activity of the extract in HT-29 human colon cancer cells [9].

*Hemidesmus indicus* (L.) R.Br. (*Apocynaceae*)—known also as the vernacular name of Indian sarsaparilla—is an Indian weed of which the crude drug (root) is used in Ayurvedic medicine to cure a whole assortment of disorders and diseases such as diarrhea, dysentery, fever, inflammation, and pain [10,11]. Systematical studies about the pharmacological profile of *H. indicus* begun more than 50 years ago, and many current in vitro, in vivo, and ex vivo studies, explained some traditional uses and explored the antitumor potential of *H. indicus* alcoholic or aqueous root extracts on different cancers, such as leukemia, breast, hepatic, colon, and skin cancer [12,13,14]. For example, a root decoction of *H. indicus* showed anti-angiogenic effects and cytotoxic and cytostatic properties on promyelocytic leukemia cells, while it triggered the apoptotic mitochondrial pathway on T-lymphoblastic cells [15,16]. In vivo, a topical application of an all-plant extract in Swiss albino mice prevented the 7,12-dimethyl-benz[a]anthracene-mediated initiation and the 12-*O*-tetradecanoyl 13-phorbol acetate-driven promotion of skin carcinogenesis [13].

The aim of this study was to evaluate the cancer chemopreventive properties and expand the knowledge about the therapeutic potential of *H. indicus* using a particular hydro-alcoholic root extract on an acute lymphoblastic leukemia cell line (CCRF-CEM). The extract employed in the study differed from other tested preparations since, after the first hydro-alcoholic extraction, it has undergone a further soxhlet passage. The result was an extract enriched in 4-hydrobenzaldehyde (4HB), in addition to the three usual main phytomarkers of *H. indicus* root extracts: 2-hydroxy-4-methoxybenzaldehyde (2H4MB), 3-hydroxy-4-methoxybenzaldehyde (3H4MB), and 2-hydroxy-4-methoxybenzoic acid (2H4MBA) [16]. Through an integrated experimental approach, we explored the genoprotective potential of the soxhlet *H. indicus* extract (HIHE soxhlet) against different mutagenic compounds and its cytotoxic, proapoptotic, and cytostatic properties.

## 2. Results and Discussion

The RP-HPLC-DAD analyses identified and quantified 4-hydrobenzaldehyde (4HB), 2-hydroxy-4-methoxybenzaldehyde (2H4MB), 3-hydroxy-4-methoxybenzaldehyde (3H4MB), 2-hydroxy-4-methoxybenzoic acid (2H4MBA), and the vanillic acid (4H3MBA) (Table 1). Protocatechuic acid and syringic acid, in this order of elution, were identified but not quantified because their concentrations were under the limit of quantitation (LOQ) of the instrument. 2H4MB was the main component with a relative presence of more than 77%, followed by 3H4MB (10%), 4HB (4.5 %), and 2H4MBA (4%) of the total phytocomplex. The soxhlet extraction was carried out six times, and the analyses of the chemical fingerprinting were performed on every sample. Results indicated no significant differences among the samples in terms of phytomarkers content.

**Figure 1:**
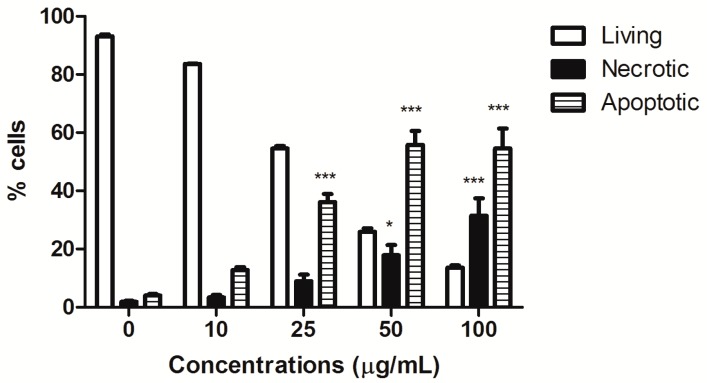
Percentage (%) of living, apoptotic, and necrotic cells after 24 h treatment of CEM cells with increasing concentrations of HIHE soxhlet. * *p* < 0.05; *** *p* < 0.001 versus untreated cells.

**Figure 2:**
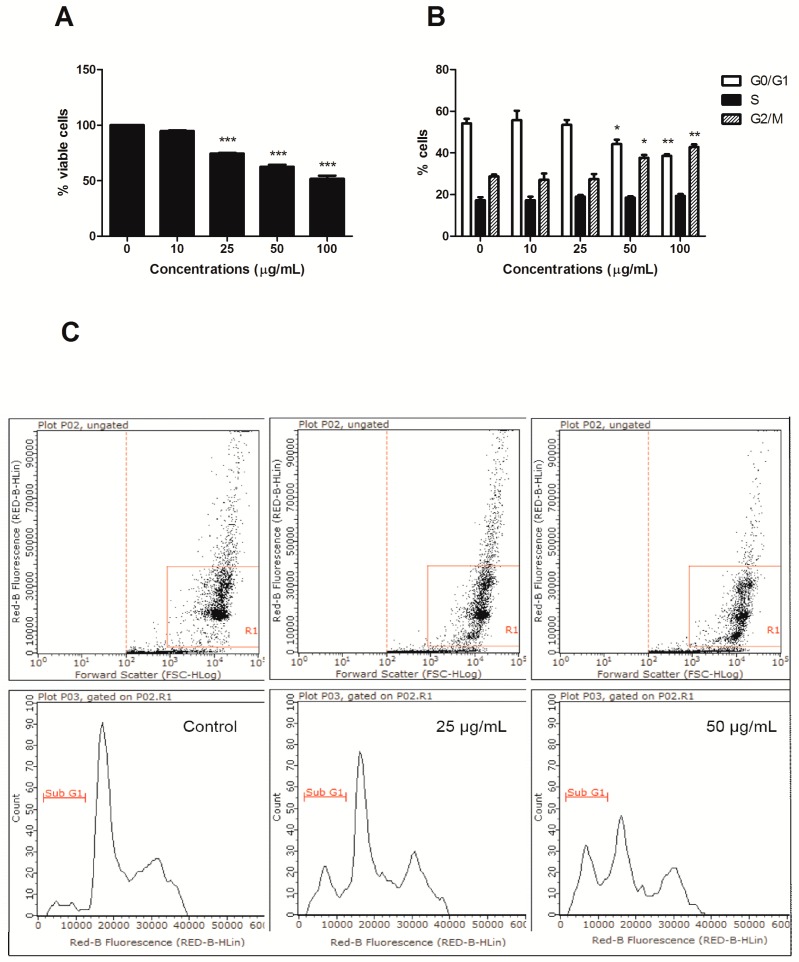
Percentage (%) of viable cells (**A**), cell-cycle distribution (**B**), and % of apoptotic cells (**C**) after 24 h treatment of DLD1 cells with increasing concentrations of HIHE soxhlet. * *p* < 0.05; ** *p* < 0.01; *** *p* < 0.001 versus untreated cells.

HIHE soxhlet induced a significant increase in the fraction of apoptotic cells starting from the concentration of 25 µg/mL (36.15% versus 4.05% of untreated cells), and the percentage of apoptotic cells further increased at higher tested concentrations (50 µg/mL: 55.80% apoptotic cells). However, starting from this concentration, the fraction of necrotic cells was also significantly increased (17.91% at 50 µg/mL and 31.46% at 100 µg/mL, respectively, versus 1.86% of the control), even if the necrotic fraction was much lower than the apoptotic one (Figure 1). Of note, HIHE soxhlet induced similar effect on cell viability on colorectal adenocarcinoma DLD1cells, in which we observed an increase in the fraction of apoptotic cells (i.e., sub-G1 cells) (Figure 2A,C).

HIHE soxhlet significantly decreased cell viability of T-lymphoblastic cells, and its cytotoxic activity depends on its ability to increase the apoptotic cell fraction. The apoptotic process could be mediated by two different pathways: the intrinsic/mitochondrial pathway and/or the extrinsic/receptorial pathway [17]. In the mitochondria, different signals converge for the activation of apoptotic cells death. These signals include cytochrome c release, loss of transmembrane potential, and alteration of cellular redox pathway [18]. Transmembrane potential loss, release of apoptogenic factors, and loss of oxidative phosphorylation are induced by the opening of the pores of the mitochondrial permeability transition and lead to intrinsic apoptosis, which is regulated by members of the Bcl-2 family [17,18]. Fas ligand is involved in the apoptotic extrinsic pathway. It binds its FADD receptor, leading to the cell death complex formation that includes pro-caspases 8 and 10. The activation of caspases 8 and 10 finally leads to caspases 3 and 7 activation, resulting in apoptotic cell death [19].

**Figure 3:**
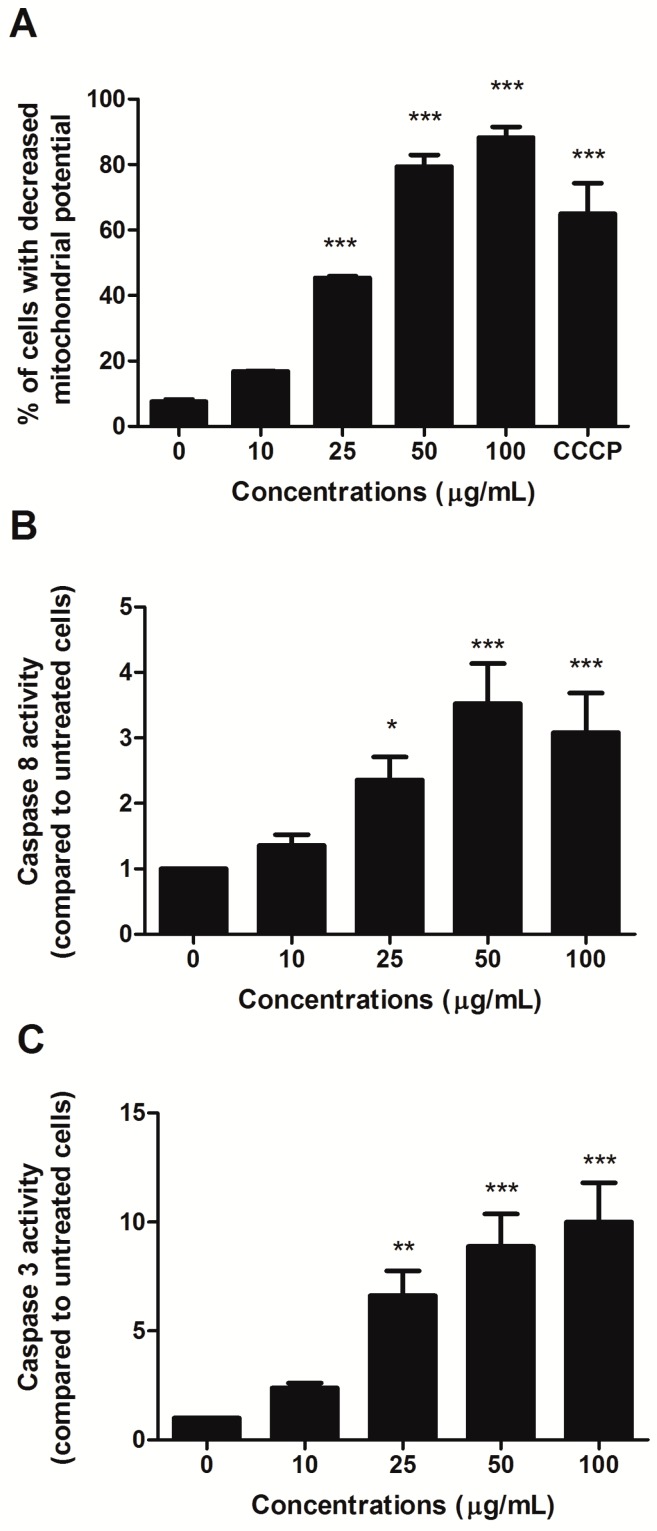
Percentage (%) of cells with decreased mitochondrial potential (**A**), and caspase 8 (**B**) and caspase 3 (**C**) activities after 24 h CEM treatment with increasing concentrations of HIHE soxhlet. * *p* < 0.05; ** *p* < 0.01; *** *p* < 0.001 versus untreated cells. CCCP: carbonyl cyanide 3-chlorophenylhydrazone.

To investigate the involvement of the intrinsic pathway in the proapoptotic activity of HIHE, we analyzed the mitochondrial transmembrane potential of treated cells. A significant decrease in the mitochondrial potential was recorded starting from 25 µg/mL treatment (45.39% of cells with decreased mitochondrial potential versus 7.65% in untreated cells) (Figure 3A). At higher concentrations, the decrease was even higher than that observed for the positive control CCCP (88.3% for cells treated with 100 µg/mL HIHE soxhlet versus 64.98% for cells treated with CCCP 50 µM) (Figure 3A).

Additionally, the activity of caspase 8 and caspase 3 was evaluated. Their activities significantly increased after 24 h from HIHE soxhlet treatment and at 25 µg/mL resulted in 2.36- and 6.62-fold increases, respectively, compared to untreated cells. Caspase 8 reached its maximum activity at 50 µg/mL with 3.52-fold increase, and caspase 3 at 100 µg/mL with 10.01-fold increase (both values compared to control (Figure 3B,C). Caspase 3 activation was further confirmed by the up-regulation of cleaved poly ADP-ribose polymerase (PARP), an important reporter of caspase-3 activity (data not shown).

In our experimental conditions, both intrinsic and extrinsic pathways were activated. HIHE soxhlet decreased the transmembrane mitochondrial potential and increased caspase 8 and caspase 3 activities. Of note, the activation of caspase 3 was several folds higher compared to that of caspase 8. This is not surprising, because caspase 3 is activated by both intrinsic and extrinsic apoptotic pathways [17,18]. Furthermore, the overexpression of cleaved PARP, a key reporter of caspase 3 activation, confirmed this evidence [20]. Interestingly, previous results obtained with *H. indicus* root traditional preparation (decoction) in another T-lymphoblastic cell line (Jurkat) showed the activation of intrinsic pathway, but no modulation of the extrinsic one [16]. This difference could be attributed to the composition of the hydro-alcoholic extract, in which the organic components are more concentrated than in the decoction. All the molecules identified in the extract are derived by the shikimic acid metabolism pathway, and possess cytotoxic activity in different cancer cell lines [12,21,22]. Comparing the concentrations of 2H4MB, 3H4MB, and 2H4MBA of the soxhlet extract with those of the decoction and hydroalcoholic extract of *H. indicus* [12,16], we observed a much higher concentration of these compounds in the extract than that detectable in the decoction and hydroalcoholic extract. As an example, the concentration of 2H4MB was 266.4489 ± 6.7318 mg/g of dried extract obtained with soxhlet, 0.0806 ± 0.0032 mg/g of dried extract obtained by decoction, and 8.790 ± 0.0021 mg/g of dried extract obtained by hydroalcoholic extraction. Moreover, molecules that were under the detection limit in the previous formulation (e.g., 4H3MBA) were detectable and quantifiable in the soxhlet extract. The punctual fractionation of successive extracts of *H. indicus* represents a strategy to increase its bioactivity. In light of the solubility of the over mentioned compounds (freely soluble in the ethanol/chloroform mixture and barely soluble in water) [23], soxhlet extraction was used to minimize the sugar presence and to increase the concentration of bioactive metabolites, as previously demonstrated for *H. indicus* decoction [15]. In parallel to these considerations, the low solvent consumption of this technique was another advantage that made us opt for the choice of this method.

**Figure 4:**
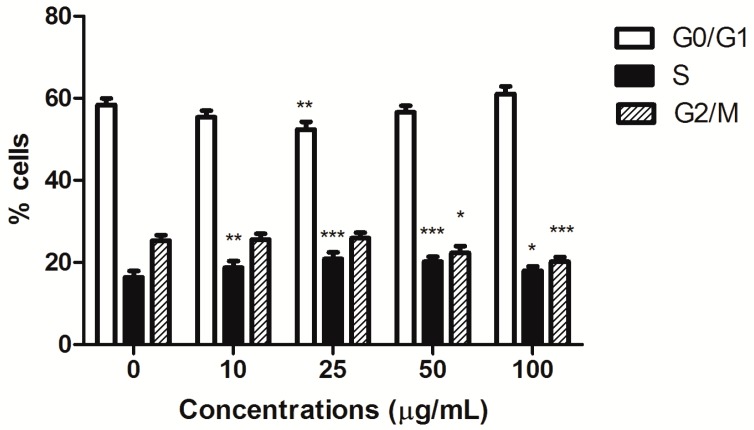
Cell-cycle distribution of CEM cells after 24 h treatment with HIHE soxhlet. * *p* < 0.05; ** *p* < 0.01; *** *p* < 0.001 versus untreated cells.

Furthermore, HIHE soxhlet induced a cell-cycle block. In CEM cells, the block was recorded in S phase starting from the lowest tested concentrations (from 16% of untreated cells to a maximum of 21% for treated cells), complemented by a slight compensatory decrease in the G0/G1 phase at the concentration 25 µg/mL (52.37% versus 58.33% of untreated cells) and in the G2/M phase for higher tested concentrations (20.16% versus 25.29% of control at the highest tested concentration) (Figure 4).

**Figure 5:**
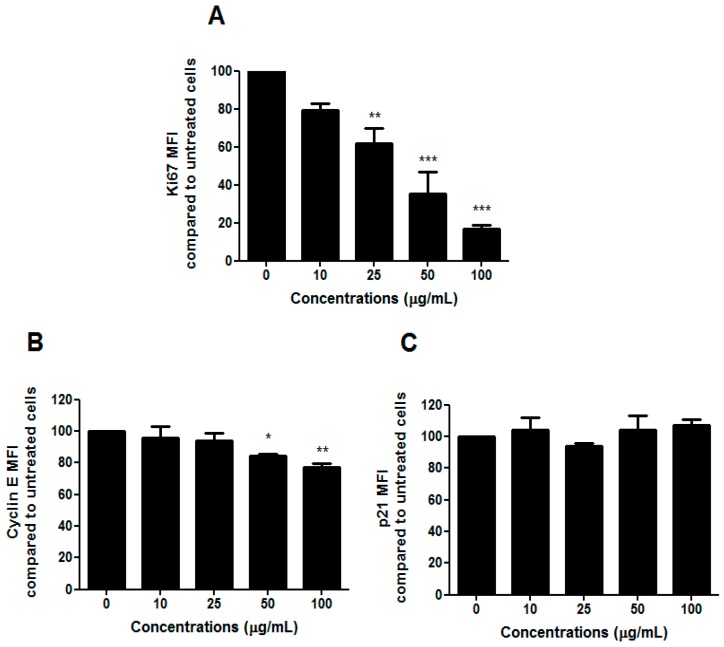
KI67 (**A**), cyclin E (**B**), and p21 (**C**) expression of CEM cells after 24 h treatment with HIHE soxhlet. * *p* < 0.05; ** *p* < 0.01; *** *p* < 0.001 versus untreated cells.

To understand whether the slight change in the cell-cycle progression we observed after treatment with HIHE soxhlet was associated with an inhibitory effect on cell proliferation, we analyzed the expression of the human Ki67 protein. Due to its presence during the active phases of the cell cycle, but its absence in resting cells, Ki67 represents an excellent marker for cell proliferation [24]. We recorded a marked decrease in Ki67 expression (Figure 5A).

Cells usually undergo cell-cycle arrest in G1/S or G2/M phase, and cell-cycle check-point from G1 to S phase represents a critical cellular event. The ability of HIHE soxhlet to inhibit cell cycle in S phase, where DNA replicates, could represent an interesting pharmacological strategy in a critical point for the neoplastic transformation [25]. Moreover, cyclin-CDK complexes exert a key role in G1-S checkpoint. A previous study on *H. indicus* root decoction already showed a S-phase delay, accompanied by a down-regulation of CDK2 that, despite an increase in cyclin E, finally led to cell-cycle block mediated by p21, gene responsible for cell-cycle arrest at the G1–S transition [16,26]. In the present study, the inhibition of cell-cycle progression was supported by a modulation of cyclin E (Figure 5B), while p21 was not affected by HIHE soxhlet (Figure 5C).

The cytostatic effect of HIHE soxhlet was highlighted also on DLD1 cells, where we observed a cell-cycle block in G2/M phase (Figure 2B).

On the whole, our results suggest that *H. indicus* evokes an inhibition of cell proliferation supported by a cell type-dependent modulation of a different phase of the cell cycle.

**Figure 6:**
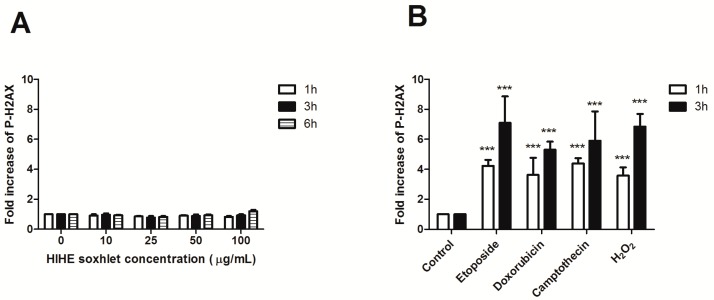
Relative expression of P-H2AX after treatment with HIHE soxhlet (**A**), etoposide 10 µg/mL, doxorubicin 5 µM, camptothecin 5 µM, or H_2_O_2_ 1 mM (**B**). *** *p* < 0.001 versus untreated cells (control).

To investigate the genoprotective potential of HIHE soxhlet, we performed a preliminary experiment to exclude its genotoxicity. The phosphorylation of H2AX (P-H2AX) was analyzed after 6 h from treatment up to 100 µg/mL. No significant increase in P-H2AX was observed at any tested concentration and at any time point, thus excluding any genotoxic activity of the extract (Figure 6A).

Although DNA damage in both somatic and germinal cells can lead to serious health problems, evidence of the DNA damage ability of multicomponent botanical products is scanty, as opposed to single isolated phytochemicals. This is true also for *H. indicus*. As an example, 2H4MB and 3H4MB were found to be genotoxic at 9–152.15 mg [27]. Additionally, Erdem et al. (2012) observed that 12 μM 4H3MBA (corresponding with about 1.83 mg) was genotoxic to human lymphocytes using the micronucleus test and comet assay, while a concentration of 6 μM (corresponding with about 0.91 mg) was not genotoxic [28]. In contrast to those results, we did not observe any genotoxic potential for HIHE. The reason why we did not record a genotoxic potential for *H. indicus* root extract can be attributed to the peculiar nature of botanical products, in which matrix effects should be considered. The plant matrix—mainly characterized by wall components as cellulosic material, hetero-polysaccharides, pectins, etc.—can actually cause a slow or incomplete release of key components. Moreover, other phytochemicals of the extract can synergize or antagonize the biological effect of a single phytochemical or modify pH and texture of the matrix [29,30]. As an example, ferulic acid in whole grain wheat binds with high affinity to polysaccharides of the plant matrix, leading to a reduction of its release and a low bioaccessibility (<1%) [31]. Accordingly, different studies indicated that the results obtained with an extract containing genotoxic compounds in its plant matrix can differ from those recorded when using the isolated genotoxic compound [32].

We then investigated the ability of the extract to protect cells from the genotoxic activity of different mutagens: H_2_O_2_, an oxidizing agent that induces DNA single- and double-strand breaks [33]; etoposide, which targets DNA topoisomerase II activities and leads to the production of DNA breaks [34]; doxorubicin, a benzanthroquinone anticancer agent with intercalates DNA and exhibits both clastogenic and aneugenic effects [35]; and camptothecin, which inhibits topoisomerase I and prevents DNA religation [36]. Mutagens were tested after 1 and 3 h of treatment. After 1 h incubation, P-H2AX showed a 4.2-fold increase for etoposide, 3.6-fold increase for doxorubicin and H_2_O_2_, and 4.4-fold increase for camptothecin (Figure 6). The phosphorylation further increased after 3 h of mutagens exposure (7.1 for etoposide, 5.3 for doxorubicin, 5.9 for camptothecin, and 6.8 for H_2_O_2_), whereas the lack of increase in P-H2AX was confirmed for HIHE soxhlet (Figure 6).

**Figure 7:**
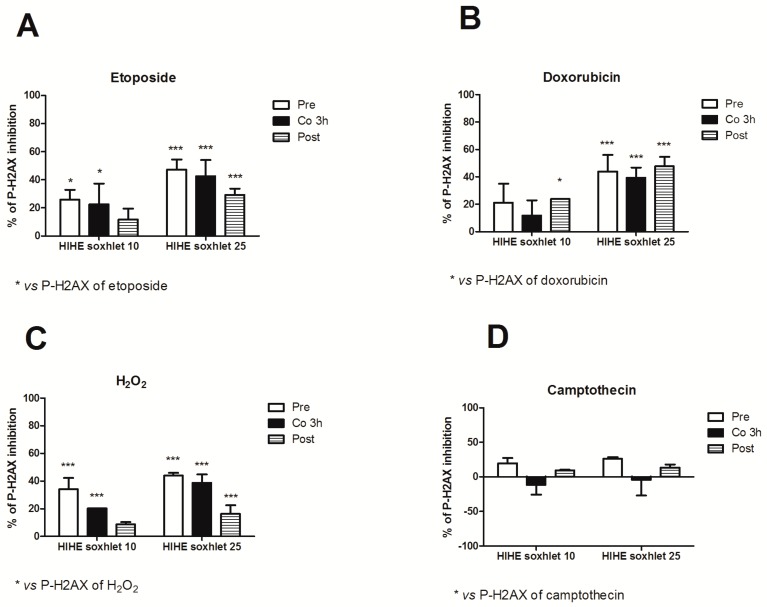
Percentage (%) of P-H2AX inhibition 1 or 3 h after pre-, co-, and post-treatment with HIHE soxhlet 10 and 25 µg/mL and etoposide (**A**), doxorubicin (**B**), H_2_O_2_ (**C**), or camptothecin (**D**). * *p* < 0.05; *** *p* < 0.001 versus P-H2AX of etoposide (**A**), doxorubicin (**B**) or H_2_O_2_ (**C**).

We used three different experimental designs (pre-, co-, and post-treatment) to explore the genoprotective activity of *H. indicus* root extracts and explore the mechanisms of its antigenotoxic potential. HIHE soxhlet strongly inhibited the genotoxicity of etoposide in all the experimental conditions. In particular, at the highest tested concentration (25 µg/mL), HIHE determined a reduction up to 47.3%, 42.6%, and 29.2% in the phosphorylation of H2AX in the pre-, co-, and post-treatment, respectively (Figure 7A).

When HIHE soxhlet was tested for its genoprotective effect against doxorubicin-mediated genotoxicity, the extract reached a 23.9% inhibition at 10 µg/mL and a 47.8% inhibition at 25 µg/mL in the post-treatment protocol (Figure 7B). The pre- and 3 h co-treatment protocols resulted in a significant reduction of H2AX phosphorylation only at the highest tested concentration (43.9% and 39.5%, respectively) (Figure 7B).

On the other hand, HIHE soxhlet led to a significant decrease in P-H2AX induced by H_2_O_2_ in all three experimental protocols (Figure 7C). The highest protective effect was evidenced in the pre-treatment protocol and at the highest tested concentration (25 µg/mL), where the phosphorylation of H2AX was reduced of 43.9%. Similar results were obtained after 3 h of co-treatment H_2_O_2_/HIHE soxhlet 25 µg/mL (38.7% of inhibition). A lower but significant genoprotection was also observed following the post-treatment protocol, but only at the highest tested concentration (16.4% of inhibition) (Figure 7C).

In all treatment protocols, there was a standard concentration-response relationship, since higher concentrations showed a higher genoprotective activity against DNA damage induced by etoposide, doxorubicin and H_2_O_2_.

Under our experimental conditions, only the pre-treatment condition slightly modulated the genotoxicity of camptothecin. Neither the co-treatment nor the post-treatment affected its genotoxicity, and the value observed for each experimental condition was similar or even higher than that of camptothecin-treated cultures (Figure 7D).

Of note, for all the genotoxic compounds we tested, in the genoprotective potential of the extract also after 1 h of co-treatment protocol, but only for H_2_O_2_, we observed a significant reduction of the DNA damage (data not shown).

The genoprotective ability of *H. indicus* root extract we demonstrated in this paper is in line with an in vitro and in vivo study showing that the aqueous and ethanolic extracts protect against cisplatin genotoxicity [37,38].

DNA protective substances can act as desmutagenics or bioantimutagenics. Desmutagenics prevent DNA damage by irreversibly binding to the genotoxic agent or its precursor, in intra- or extracellular compartment, and promoting its chemical inactivation through the modulation of phase 1 and phase 2 enzymes [39]. Desmutagenic agents can also act as antioxidants and radical scavengers [40]. Bioantimutagens act within the cell by preventing the fixation processes (i.e., DNA replication) of DNA damage and/or promoting its protection and repair [39]. According to our results and taking into account the experimental strategy used, the antigenotoxic activity of HIHE probably involves both desmutagenic (pre- and co-treatments) and bioantimutagenic (post-treatment) mechanisms. Thus, *H. indicus* extract could act (1) at extra-cellular level by reducing the rates of absorption and uptake of the genotoxic agent; and (2) at extra- and intra-cellular level by complexing the genotoxic agent and/or modulating DNA repair, cell cycle, or apoptosis.

The observation of a dose-effect relationship for all the anti-genotoxicity effects may be due to its ability to act via similar mechanisms including antioxidant mechanisms. Plant extracts may contain a variety of chemical compounds including polyphenols, flavonoids, vitamins, and carotenoids, through which they afford cytoprotective properties, as well as the ability to block free radicals, the modulation of apoptosis and cell cycle, and the ability to produce cytokines and activate DNA repair. One cytoprotective mechanism weakening the genotoxicity of different compounds and promoting the organism’s resistance to genotoxicants is the antioxidant activity. Some types of chemical genotoxics induce oxidative stress. In this condition, critical molecular targets such as genes and proteins are exposed to free radical attack.

As 2H4MB was the most abundant component (84.76%) of our extract, the genoprotective activity of the root extract of *H. indicus* could be mainly attributed to this compound. 2H4MB was screened for its antioxidant activity by three complementary tests, obtaining the following median inhibitory concentrations: 9.04 mg/mL in the 2,2-diphenyl-1-picrylhydrazy free radical scavenging test and 0.25 mg/mL in the β-carotene-linoleic acid test. 2H4MB was also found to exhibit chelating activity determined by the ferrozine assay, in which the median inhibitory concentration was 2.31 mg/mL [41]. The genotoxicity of H_2_O_2_ and, at least in part, that of doxorubicin can be attributed to the production of free radicals. However, doxorubicin can also act by intercalating into the DNA strand and inhibiting DNA topoisomerase II [42]. This evidence suggests that mechanisms other than the antioxidant can be involved in the genoprotective activity of *H. indicus* extract. The ability of *H. indicus* phytocomplex to markedly reduce the genotoxicity of etoposide, a topoisomerase II inhibitor, supports this hypothesis.

The evidence that treatment with *H. indicus* extract increased the genotoxicity induced by camptothecin indicates that such results might be related to the presence of chemical components in *H. indicus* samples that interact with camptothecin or its targets at the time of damage induction. It is important to note that the methanolic extract of *H. indicus* root was found to partially inhibit human topoisomerase I at 40 μg/mL and partially inhibit human topoisomerase II at 120 μg/mL [43]. Taking into account the concentrations of *H. indicus* extract tested in our antigenotoxic study (10 and 25 μg/mL), the inhibition of topoisomerase II could not take place in our experimental settings, while the inhibition of topoisomerase I could. All in all, we think this would explain the reason why *H. indicus* phytocomplex protects against the genotoxicity of etoposide while it potentiates the genotoxicity of camptothecin.

## 3. Conclusions

As the world’s population is constantly aging and chronic diseases such as cancer have become more prevalent, drug development shifted its focus towards not only treating but preventing chronic diseases. Botanical drugs are very complex sources of bioactive substances that can act on different “druggable” targets. In this paper, we report on the wide spectrum of therapeutic potential against cancer, as well as on the in vitro cancer chemopreventive activity of *H. indicus* hydro-alcoholic root extract. HIHE soxhlet induces apoptosis and cell-cycle inhibition in human leukemia cells and colon adenocarcinoma cells, and exhibits genoprotective activity against mutagens acting through a different DNA damage mechanism. These data add to the growing body of evidence that natural compounds can represent a noteworthy strategy for targeting early and late stages of cancer.

## 4. Materials and Methods

### 4.1. Plant Extract Preparation

*H. indicus* (Batch No. 3904, Mgf Date 24/08/2011) were collected from Ram Bagh (Amritsar Punjab, Rajasthan, India), authenticated by Dr. MR Uniyal, Maharishi Ayurveda Product Ltd., Noida, India. After harvesting, the roots were cleaned and cut into small pieces before being dried. All samples were then ground to a fine powder and kept at −20 °C until used for the extractions. HIHE preparation followed the procedure reported elsewhere [44]: briefly, 50 g of ground root were mixed with 450 mL of 30% ethanol–water solution. Mixtures were left for 21 days and were stirred constantly. Extracts were then filtered, lyophilised, and stored at −20 °C. All the extractions were performed in triplicate. Immediately before use, the samples were resuspended in 30% ethanol–water solution.

Soxhlet extraction was performed in order to extract non-volatile and semivolatile organic compounds from dried HIHE, and concentrate the organic compounds present in the phytocomplex. An aliquot of 2 g of solid sample was placed in a 30 mL soxhlet apparatus under reflux conditions with 40 mL of a 1:1 mixture of CHCl_3_ and ethanol (heating mantle set to 80 °C) for 6 h. Subsequently, 10 mL of ddH_2_O were added to the extraction mixture in a separation fennel, and two phases were collected and dried.

### 4.2. HPLC Analysis

*H. indicus* fractions obtained by soxhlet extraction were subjected to RP-HPLC-DAD analysis to identify and quantify their main phytomarkers. The analysis was performed using a Jasco modular HPLC (model PU 2089, Tokyo, Japan) coupled to a diode array apparatus (MD 2010 Plus) linked to an injection valve with a 20 µL sampler loop (40 µL injection volume). The system was composed of a Kinetex XB C18 (5μm, 15 × 0.46 cm, Phenomenex, Aschaffenburg, Germany) column, with a flow rate of 0.7 mL/min, a mobile phase consisting of two solvent solutions, A (water/formic acid, 99.9:0.1) and B (methanol), combined in a gradient system characterised by a starting point (0 “starting from”) of 5% B, which rose to 30% in 50 min, followed by an isocratic phase until 65 min; percentage of B fell to 5% in 5 additional min and came back to the initial condition in 10 min. All solvents used were of chromatographic grade (Sigma Aldrich, St. Louis, MI, USA). The recorded chromatograms of the phytocomplexes were compared to those of pure standards in terms of UV absorption and retention time.

### 4.3. Cell Culture and Treatment

The T-lymphoblastic cell line CCRC-CEM and human colon adenocarcinoma cell line DLD1 were purchased from LGC Standard (LGC Group, Middelsex, UK). Both cell lines were grown in Roswell Park Memorial Institute (RPMI) 1640 (Sigma Aldrich), supplemented with 10% heat-inactivated bovine serum (Gibco, Life Technologies, Monza, Italy), 1% penicillin/streptomycin solution, 1% l-glutamine solution and, only for CCRC-CEM, with 1% sodium pyruvate solution (all obtained from Biochrome, Merck Millipore, Darmstadt, Germany). Cells were incubated at 37 °C with 5% CO_2_. To maintain exponential growth, CCRC-CEM were cultured at the density of 2 × 10^6^ mL. DLD1 were trypsinized when they reached 70–80% confluency.

Cells were treated with increasing concentration of HIHE soxhlet (0–100 μg/mL) for 1, 3, 6, or 24 h according to experimental requirements. Etoposide 10 µg/mL, doxorubicin 5 µM, camptothecin 5 µM, H_2_O_2_ 1 mM, and carbonyl cyanide 3-chlorophenylhydrazone (CCCP) 50 µM were used as positive controls.

### 4.4. Analysis of Cell Viability and Detection of Apoptosis

Guava ViaCount Reagent (Merck Millipore, Billerica, MA, USA) was used to evaluate cells’ viability of CCRC-CEM cells. Briefly, cells were diluted with the reagent containing 7-amino-actinomycin D (7-AAD) and incubated at room temperature in the dark for 5 min before flow cytometric analysis. The IC_50_, the concentration that inhibits cell viability of 50%, was calculated by interpolation from dose-response curve. Concentrations ≤IC_50_ were used in the subsequent experiments.

Necrotic and apoptotic events were discriminated using Guava Nexin Reagent (Merck Millipore), which contains 7-AAD and annexin V-phycoerythrin. Cells were incubated for 20 min at room temperature in the dark and samples analyzed via flow cytometry.

To measure the cell viability of DLD1 cells, intracellular alkaline esterase activity was measured through the 4-methylumbelliferyl heptanoate (MUH) assay (Sigma Aldrich), according to manufacturer’s recommendations. Briefly, cells were seeded in a 96-well plate and, after 24 h treatment with HIHE soxhlet, were incubated with 1 mg/mL MUH diluted in PBS 1X for 20 min at 37 °C. The fluorescence was measured by the plate reader Victor X3 (Perkin Elmer, Walthman, MA, USA) at 330 nm excitation and 450 nm emission.

### 4.5. Cell-Cycle Analysis

After 24 h from treatment with HIHE soxhlet, cells were fixed and permeabilized with 70% ice-cold ethanol, washed with PBS 1X, and incubated with 200 µL of Guava Cell Cycle Reagent (Merck Millipore) containing propidium iodide. Samples were incubated in the dark at room temperature for 30 min and analyzed via flow cytometry.

### 4.6. Analysis of PARP, Ki67, Cyclin E, and p21 Expression

After treatment with HIHE soxhlet for 24 h, cells were fixed by 4% paraformaldehyde in PBS 1X and permeabilized using 90% cold methanol. Samples were then incubated with the fluorescein isothiocyanate antibody of cleaved 85 kDa fragment PARP (1:100, Invitrogen, Carlsbald, CA, USA), Ki67 Alexa Fluor 488 (Abcam, San Francisco, CA, USA), cyclin E (1:10, Abcam), or p21 (1:10, Abcam). Cells incubated with cyclin E or p21 were washed and then incubated with fluorescein isothiocyanate-labeled secondary antibody (1:100, Sigma Aldrich). All samples were analyzed via flow cytometer and mean fluorescence intensity (MFI) values were recorded.

### 4.7. Measurement of Mitochondrial Potential

Mitochondrial membrane potential was assessed using MitoProbeTM DilC1(5) Assay kit (Molecular Probes, Thermo Fisher Scientific, Waltham, MA, USA), according to manufacturer’ instructions. DilC1(5) (1,1′,3,3,3′,3′-hexamethylindo dicarbo-cyanine iodide) freely accumulates in mitochondria with active membrane potential. Briefly, after 24 h from HIHE soxhlet treatment, 10^6^ cells were washed and supplemented with 50 nM DilC1(5) for 20 min at 37 °C, 5% CO_2_. Cells were washed with PBS 1X and suspended again in PBS for flow cytometric analysis. CCCP was used as positive control. Results were expressed as % of cells with decreased mitochondrial potential compared to untreated cells.

### 4.8. DNA Damage Assay

Phosphorylation of H2AX (P-H2AX) was used as marker of genotoxicity using FlowCellectTM Histone H2AX Phosphorylation Assay Kit (Merck Millipore).

For the analysis of the genotoxic potential of HIHE soxhlet, cells were treated for 6 h at concentrations up to 100 µg/mL and then analyzed for P-H2AX phosphorylation.

**Figure 8:**
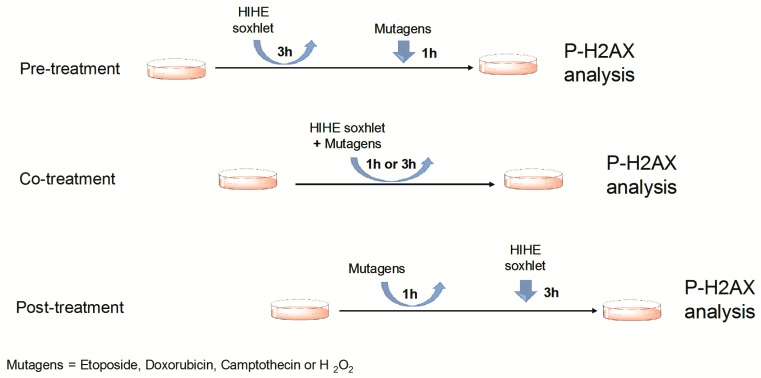
Schematic description of the experimental design used for the study of the antigenotoxic potential of HIHE soxhlet.

For the analysis of its genoprotective potential, cells were treated with HIHE soxhlet before (pre-treatment protocol), during (co-treatment protocol), or after (post-treatment protocol) the treatment with the mutagen (Figure 8):

- In the pre-treatment protocol, the cells were treated with HIHE soxhlet 10 or 25 µg/mL. After 3 h, the culture medium was removed and cells were treated with the mutagen. After 1 h, cell samples were analyzed.
- In the co-treatment protocol, cells were treated contemporarily with HIHE soxhlet 10 or 25 µg/mL plus the mutagen for 1 or 3 h; then, cell samples were analyzed.
- In the post-treatment protocol, cells were first exposed to each mutagen for 1 h, then the mutagen was removed and cells were treated with HIHE soxhlet extract for 3 h before cell sample analysis.

For H_2_O_2_ treatment, cells were treated with medium without bovine serum.

After the treatment with HIHE soxhlet and genotoxic compounds, cells were fixed and permeabilized. Samples were then incubated with an anti-P-H2AX-Alexa Fluor antibody, 1:500 (Merck Millipore) for 30 min in the dark. The analysis was performed via flow cytometry. For each mutagen compound, three different experiments were performed.

### 4.9. Evaluation of Caspase 8 and Caspase 3 Activity

Caspase activity was assessed using Caspase 8 Colorimetric Protease Assay Kit or Caspase 3 Colorimetric Protease Assay Kit, respectively (both purchased by Thermo Fisher Scientific, Carlsbald, CA, USA), according to manufacturer’s instructions. Briefly, after 24 h treatment of 3 × 10^6^ cells/sample with HIHE soxhlet, cells were washed in PBS 1X and suspended in Cell Lysis Buffer on ice for 10 min. Cellular lysates were centrifuged, collected, and normalized in terms of protein concentration, according to Bradford assay [45]. Cellular lysates were then incubated for 2 h at 37 °C in the dark with 2X Reaction Buffer, containing DTT 10 mM and 200 µM of caspase 8 or caspase 3 substrate. Both substrates consist of a synthetic tetrapeptide, IETD (Ile-Glu-Thr-Asp) specific for caspase 8, and DEVD (Asp-Glu-Val-Asp) specific for caspase 3, conjugated with the chromophore p-nitroanilide (pNA). In presence of caspases’ activity, the specific substrate is cleaved from the chromophore and free pNA is used as a reporter, whose absorbance is measured at 405 nm, using the microplate reader Victor X3 (Perkin Elmer). Caspase activity was expressed as the fold increase of treated cells compared to untreated cells.

### 4.10. Flow Cytometry

EasyCyte 5HT (Merck Millipore) was used to perform all flow cytometric analyses. For each sample, at least 5000 events were evaluated.

### 4.11. Statistical Analysis

All experiments are expressed as the mean ± SEM of at least three independent experiments. Statistical analyses were assessed by Repeated Measures ANOVA, and Dunnett was used as a post-test, using the statistical software GraphPad InStat 5.0 version (GraphPad Prism, San Diego, CA, USA). *p* < 0.05 was considered significant.

## Figures and Tables

**Table 1:** Quantification of 2-hydroxy-4-methoxybenzaldehyde (2H4MB), 3-hydroxy-4-methoxybenzaldehyde (3H4MB), 2-hydroxy-4-methoxybenzoic acid (2H4MBA), vanillic acid (4H3MBA), and 4-hydroxybenzaldehyde (4HB).

Compound	λ_analysis_ (nm)	Amount (mg/g of Dried Extract)	LOD (µg/mL)	LOQ (µg/mL)	*R*^2^
2H4MB	280	266.4489 ± 6.7318	0.0110 ± 0.0012	0.0368 ± 0.0039	0.9998
3H4MB	280	28.4669 ± 1.1387	0.0110 ± 0.0010	0.0368 ± 0.0030	0.9990
2H4MBAc	260	13.8240 ± 0.7161	0.0105 ± 0.0013	0.0351 ± 0.0034	0.9999
4H3MBAc	260	2.6517 ± 0.0661	0.0029 ± 0.0004	0.0097 ± 0.0011	0.9991
4HB	280	2.9478 ± 0.0400	0.0011 ± 0.0001	0.0036 ± 0.0001	0.9996

LOD: limit of detection; LOQ: limit of quantitation; *R*^2^: coefficient of determination of the calibration line.

## Abbreviations

7-AAD	7-amino-actinomycin D
CCCP	carbonyl cyanide 3-chlorophenylhydrazone
DEVD	Asp-Glu-Val-Asp
DilC1(5)	1,1′,3,3,3′,3′-hexamethylindodicarbo-cyanine iodide
HIHE soxhlet	*H. indicus* hydro-alcoholic extract followed by soxhlet extraction
4HB	4-hydrobenzaldehyde
HIHE	*H. indicus* hydro-alcoholic extract
2H4MB	2-hydroxy-4-methoxybenzaldehyde
3H4MB	3-hydroxy-4-methoxybenzaldehyde
2H4MBA	2-hydroxy-4-methoxybenzoic acid
4H3MBA	vanillic acid
IC_50_	the concentration that inhibits cell viability of 50%
IETD	Ile-Glu-Thr-Asp
MFI	mean fluorescence intensity
MUH	4-methylumbelliferyl heptanoate
PARP	poly ADP-ribose polymerase
P-H2AX	phosphorylation of H2AX
pNA	p-nitroanilide
